# Supplementary material for: Psychological distress and quality of life among Opioid Agonist Treatment service users with a history of injecting and non-injecting drug use: A cross-sectional study in Kathmandu, Nepal
Source: PLoS One. 2023 Feb 6;18(2):e0281437. doi: 10.1371/journal.pone.0281437 (PMC9901755; doi:10.1371/journal.pone.0281437)
Supplement: Attachment — Submitted filename: Psychological distress and quality of life among Opioid Agonist Treatment service.docx [file pone.0281437.s002.docx]

**Psychological distress and quality of life among Opioid Agonist Treatment service users with histories of injecting and non-injecting: A cross-sectional study in Kathmandu, Nepal**

Thank you for the opportunity to review this paper. The manuscript is well written, the topic is interesting, and the results provide important information for improving the effectiveness of the OAT program, but there is still the need to address some shortcomings.

Specific comments:

- In the title, emphasize that it is about drug use when it comes to the division of participants into injecting and non-injecting “Psychological distress and quality of life among Opioid Agonist Treatment service users with a history of injecting and non-injecting **drug use:** A cross-sectional study in Kathmandu, Nepal”
- In the introduction, the problem is nicely and chronologically explained, but the introduction should be a little more concise or shorter.
- In the methodology, it is necessary to briefly explain why you choose 5 out of the 12 cities where the OAT programs were implemented, and whether the programs in those cities differ from the others in terms of content. This is important because of the external validity of the study and the generalizability of the results.
- It is not recommended to start sentences with abbreviations
- Have you used the Kolmogorov - Smirnov test to test the normality of the data distribution, in case the data distribution is not normal it is necessary to use non-parametric tests.
- Start the results with a description of the sample, not with the sentence "Table 1 summarizes the socio-demographic characteristics of the OAT service user……". This sentence should be placed after the textual description of the contents of Table 1.
- In the tables, it is necessary to express one p-value for comparing the value of one independent categorical variable between injecting and non-injecting drug users, for example, for the variable education we have three p-values instead of one. It is necessary to check that the same error is not repeated in the tables below. P value is expressed to three decimal places, and values less than 0.001, e.g. 0.000 is displayed as 0.001
- In line 231, explain the abbreviation SD.
- In line 240 the sentence "Regarding SES 70 (61.9%) belonged to upper SES as 241 compared to 54(45.8%) non IDU (p=0.014)" is not clear.
- A cross-sectional study design does not allow the use of words such as predictor or risk factor because we cannot prove causality, but instead the construct "factor associated with" can be used.
- In line 325 “The possible reasons for the gender differences may be due to higher level of perceived stigma, and barriers in access to health care facilities and treatment including OAT for females”…. Are there other gender differences that could be the cause of the obtained results, maybe differences in employment, income...what do other studies say.
- In the discussion, the data from the results are often repeated, the discussion should be based more on the comparison with the results from other studies for a potential explanation of them.
